# Experiments in Vulcanizing Rubber Compounds

**Published:** 1862-06

**Authors:** George E. Hayes


Experiments in Vulcanizing Rubber Compounds.—*By George E. Hayes.*—In view of the vast amount of material manufactured and sold for rubber work, I presume, ere this, you have come to regard anything tending to improve the quality, to facilitate the process, or to explain any of the anomalous phenomena which are sometimes met with, as of general interest to the profession. I send you, therefore, the result of a few experiments on the rubber compounds, which may not be entirely without interest.

From the very first, frequent complaints have been made of the rubber sometimes coming out porous or spongy, and much speculation has been indulged in as to the cause. Some laid it to the bad quality of the rubber, others to overheating in the packing process; others, again, to the wax not being properly removed from the mould; some, to vulcanizing under water; others, again, to vulcanizing without water, other than that contained in the plaster mould. Indeed, almost any change in apparatus, or in the manipulations which could be called to mind, has been made, at times, to bear the odium.

Some time since, having occasion to vulcanize a thicker piece than usual, the oven was heated, in fifteen minutes, up to 320°, and retained there the prescribed time. On removal, the mass was found to have swelled to double its original size, and was porous inside.

The experiment was repeated under water with precisely the same result. It was then tried in one of the old-style boilers, heated up to 320° in the same time, and retained there the time directed for that apparatus—again it came out porous.

Not content to give it up thus, three coils were made five-eighths of an inch in length, and the same in diameter. One was made from the Hard Rubber Company's gum, one from the amber base, and the third was from both; the center portion being amber compound three-eighths of an inch in diameter inclosed in Rubber Company's gum, bringing the roll to five-eighths of an inch in thickness. The three pieces were vulcanized in one flask, the oven being heated up to 320° in fifteen minutes, and retained at that temperature forty minutes.

The amber was compact to its center, and perfectly vulcanized. The combined coil was also compact, both portions having apparently received just the right degree of heat and length of exposure. The Company's gum was compact to the depth of one-eighth of an inch, the central portion being spongy. This seemed to indicate one good quality at least in the amber compound, but not having a sufficient supply to test it in other respects, my attention was directed to the possibility of obtaining a better result with the Company's compound, the good qualities of which, in most respects, seems well established. When the mode of hardening rubber was first introduced to the profession, it was very natural to suppose that the Company knew, and had adopted, the best mode of procedure. All the directions from that, or any other source, so far as they came to my notice, were to heat up—no matter how rapidly—to a certain degree on the scale, and retain that heat three hours, two and a half hours, or one hour, the directions having been changed from time to time in the latter respect.

I had discovered that equally good work could be produced in much less time than the shortest above mentioned, and by exposure to no greater heat, with a modified apparatus; but it had not occurred to me that the old mode of heating up might not be the very best possible. Resolved now to test this point, a coil of the Company's rubber, three-fourths of an inch long and the same in diameter, was imbedded in plaster as usual, and subjected to a heat which was judged would be just sufficient to retain the temperature at 320°, if it had attained that degree. At the expiration of one hour the thermometer marked 210°. The flame was slightly increased. At the end of another hour it had reached 270°. Again the flame was raised very slightly, and at the expiration of the third hour it had just reached 320°. The heat was immediately removed, and the oven left to cool down to 212°, which it did in thirty minutes.

The coil was found perfectly compact, and a strip which had been put in as a test exhibited qualities which I had not before seen. It was almost malleable, could be bent with the pliers, indented with the hammer, retaining the shape given like a piece of lead, and yet it received the highest polish.

The experiment was then varied by heating up to 210° rapidly—say in ten minutes—then turning down the cut-off to the proper sized flame, to carry up the heat to 320° in two hours, and was extinguished at once on that degree being reached.

The result was the same, the time being shortened nearly one-third in the first part of the process. In a subsequent trial, a piece one-half inch in diameter came out solid, it having been heated from 210° to 320° in one hour, and the burner extinguished immediately on attaining that degree. The highest perfection seems to be attained when the thermometer marks 320° without waiting for any continued exposure. It is light colored, compact, solid and tough. A continued exposure darkens the tint, without any apparent benefit. The advantages gained by this modified process are threefold:—First, a better quality of vulcanite; second, the ability to harden thick pieces; third, the tedious watching of the thermometer to retain the heat at any one given point is not required. A very few observations only are required to insure a good result. The time may be one hour, or three, or even five, in bringing up the heat to the given point, and as the increase is so gradual, a few minutes more or less in extinguishing the burner ceases to be material. In course of these experiments, one other point has attracted my attention, which may be worthy of notice here. Almost every one has sometimes noticed a mottled appearance after polishing their vulcanite, black and tan. This has also been attributed to every conceivable cause.

On polishing the end section of many of the coils above mentioned, it was noticed that the two shades of color followed every layer of the coil, giving it the appearance of two strips of rubber rolled together, one dark, the other light. It was further observed that the light colored side was invariably the one which had been covered with the cloth, and that those pieces which had been longest exposed on the top of the pile produced the darkest shade of color, while a sheet taken from the center of the box was free from this peculiarity. It is generally supposed that the red color is given to the rubber by a mixture with it of vermilion, which is a sulphuret of mercury. Ethiops mineral is also a sulphuret of mercury, and very slight causes are sometimes sufficient to change one of these into the other, or vice versa.

It is well known that many of the compounds of mercury require to be excluded from the light to prevent a similar change of color. I throw out these suggestions as much for the benefit of those engaged in the manufacture of the rubber compounds, as to enlighten the profession on a dark subject. —*Dental Cosmos.*

